# Right Ventricular Flow Vorticity Relationships With Biventricular Shape in Adult Tetralogy of Fallot

**DOI:** 10.3389/fcvm.2021.806107

**Published:** 2022-01-21

**Authors:** Ayah Elsayed, Charlène A. Mauger, Edward Ferdian, Kathleen Gilbert, Miriam Scadeng, Christopher J. Occleshaw, Boris S. Lowe, Andrew D. McCulloch, Jeffrey H. Omens, Sachin Govil, Kuberan Pushparajah, Alistair A. Young

**Affiliations:** ^1^Department of Anatomy and Medical Imaging, University of Auckland, Auckland, New Zealand; ^2^Auckland Bioengineering Institute, University of Auckland, Auckland, New Zealand; ^3^Department of Cardiology, Auckland District Health Board, Auckland, New Zealand; ^4^Department of Bioengineering, University of California, San Diego, La Jolla, CA, United States; ^5^Department of Biomedical Engineering, King's College London, London, United Kingdom

**Keywords:** tetralogy of Fallot, 4D flow, vorticity, shape, atlas

## Abstract

Remodeling in adults with repaired tetralogy of Fallot (rToF) may occur due to chronic pulmonary regurgitation, but may also be related to altered flow patterns, including vortices. We aimed to correlate and quantify relationships between vorticity and ventricular shape derived from atlas-based analysis of biventricular shape. Adult rToF (*n* = 12) patients underwent 4D flow and cine MRI imaging. Vorticity in the RV was computed after noise reduction using a neural network. A biventricular shape atlas built from 95 rToF patients was used to derive principal component modes, which were associated with vorticity and pulmonary regurgitant volume (PRV) using univariate and multivariate linear regression. Univariate analysis showed that indexed PRV correlated with 3 modes (*r* = −0.55,−0.50, and 0.6, all *p* < 0.05) associated with RV dilatation and an increase in basal bulging, apical bulging and tricuspid annulus tilting with more severe regurgitation, as well as a smaller LV and paradoxical movement of the septum. RV outflow and inflow vorticity were also correlated with these modes. However, total vorticity over the whole RV was correlated with two different modes (*r* = −0.62,−0.69, both *p* < 0.05). Higher vorticity was associated with both RV and LV shape changes including longer ventricular length, a larger bulge beside the tricuspid valve, and distinct tricuspid tilting. RV flow vorticity was associated with changes in biventricular geometry, distinct from associations with PRV. Flow vorticity may provide additional mechanistic information in rToF remodeling. Both LV and RV shapes are important in rToF RV flow patterns.

## Introduction

Many patients with repaired Tetralogy of Fallot (rToF) survive to adulthood due to successful primary repair (1). Yet, chronic pulmonary regurgitation is often a consequence, leading to shape, hemodynamic and electrophysiological changes in the right ventricle (RV) (2, 3). Pulmonary valve replacement is often required; however, the timing of this procedure remains controversial (1). Previous studies analyzed the regional three-dimensional (3D) alterations of the RV anatomy in rToF (2–4). RV shape changes have been associated with common clinical metrics such as pulmonary regurgitant volume (PRV), indicating links between PRV and RV dilatation, with outflow tract bulging and apical dilation (3, 5). Remodeling patterns have been shown to be different in rToF patients compared with pulmonary hypertension patients (6) and quantification of these patterns has been facilitated by shape modeling (7).

3D statistical shape modeling enables quantification of the variation in cardiac shapes and their relationships with disease processes (8–10). RV shape can be represented as morphometric scores based on comparison with an atlas of cardiac shapes. These scores have stronger associations with risk factors such as diabetes and hypertension, giving more powerful quantitative shape measures, than standard measures of mass and volume (9).

Altered blood flow patterns in the heart are also associated with ventricular shape (11). Time-resolved phase-contrast cardiac magnetic resonance imaging, 4D flow MRI, has enabled the qualitative and quantitative measurement of altered flow patterns (12–14). In particular flow vorticity is an index of flow “swirl,” including circular, helical or spiral patterns, indicative of high shear in the flow field. These are indicative of higher viscous energy losses, and increased hemodynamic wall shear stress. Although increased vorticity has been described in the LV outflow tract in rToF patients (15), the relationships between biventricular shape and flow vortices is unknown. Application of vorticity estimation in rToF patients is also limited by noise inherent in the data, which is heightened by numerical differentiation required for the calculation of vorticity. Neural networks show promise in enhancing medical imaging data (16), but these methods have not yet been applied in the estimation of vorticity.

We aimed to examine relationships between biventricular geometry and flow vortices in the RV and the right ventricular outflow tract using a processing pipeline including enhancement of velocity vectors using a deep neural network, quantification of vorticity using numerical differentiation, and correlation with morphological scores calculated by projection onto an atlas of rToF patients. To our knowledge, this work is the first study investigating relationships between vorticity and ventricular shape.

## Methods

### Research Design and Patient Criteria

This pilot study comprised a prospective, cross-sectional design performed at the Centre for Advanced MRI (CAMRI) at the University of Auckland between 2019 and 2021. Ethics committee approval was obtained from the Health and Disability Ethics Committee New Zealand (17/CEN/226). Informed written consent was obtained for all patients and volunteers. The study group comprised twelve rToF patients recruited from the outpatient cardiology clinic, i.e., patients above 16 years of age attending annual follow-up by cardiovascular magnetic resonance. Ten healthy age- matched volunteers with no known cardiovascular abnormalities were imaged to evaluate flow differences with the study group.

### MRI Protocol

Cardiovascular magnetic resonance was performed on a Siemens Magnetom 1.5T Avanto Fit (Siemens Healthcare, Erlangen, Germany) MRI scanner. 2D steady state free precession scans were acquired using a multi-element cardiac coil, with breath-holds and retrospective gating. The typical set of sequence parameters included a repetition time of 2.9 ms, echo time of 1.36 ms, flip angle was 58°, field of view was 400 mm. The slice thickness was 6 mm, image matrix was 192 × 256 and 30 heart phases were reconstructed. Images covered the span of both ventricles. Long-axis slices were obtained through both ventricles and through the four valves. A stack of short-axis slices was acquired parallel to the tricuspid valve, covering all valves, and spanning both ventricles.

4D-flow MRI was acquired during free-breathing, using a navigator gated gradient-echo pulse-sequence with interleaved 3D flow-encoding and retrospective vector cardiogram controlled cardiac gating. Standard acquisition parameters included velocity encoding (VENC) of 150 cm/s, a flip angle of 7°, echo time 2.3 ms and repetition time 38.8 ms. The VENC was chosen to achieve minimal aliasing across all studies. The scan covered the whole heart, the aorta, and the main pulmonary artery at a spatial resolution of 2.4 × 2.4 × 2.4 mm. Parallel imaging was used with an image acceleration factor of 3, with a scanning time of 7.3–10 min. No contrast was used.

### Quantitative Measurements of Flow and Vorticity

Flow acquisitions were processed using 4D Flow Demonstrator V2.3 software (Siemens AG, Erlangen, Germany) after antialiasing and background phase correction. Antialiasing allowed for an increase in the range of measurements done to include flow from 0 to 300 cm/sec without the aliasing effects. Forward volume, reverse volume, and the PRV index (PRVi, volume divided by BSA) were measured for the pulmonary flow, and the tricuspid valve was examined for forward flow volume and regurgitant volume.

For vorticity analysis, the phase-contrast images were first denoised using the 4D FlowNet neural network (16). This was done to capture vortices that extended along multiple timeframes and achieve consistent results in the presence of noise, since vorticity requires the calculation of numerical derivatives which are sensitive to noise. Briefly, 4D FlowNet is a noise reduction and super-resolution residual network based on the SRGAN architecture (17). This deep learning network was trained solely using synthetic 4D Flow MR images, generated from computational fluid dynamics simulations. Although 4DFlowNet was designed to perform both denoising and super-resolution, we applied the network with an up sample ratio of 1, which effectively performed denoising without increasing spatial resolution since the flow features (intraventricular vortices) were large relative to the voxel size in our application.

Vorticity was calculated in ParaView (v5.8.0) as the curl of the velocity field (18, 19). Calculation of vorticity was performed by a first-order bilinear interpolation scheme over the chosen velocity vectors. This property enabled the generation of vorticity magnitude of the computed vector gradient tensor from velocity vector cell data. Subsequently, vorticity vector magnitudes were summed across the region of interest (ROI). Vorticity was reviewed on overlapping timeframes that included only the diastolic phase of the cardiac cycle. Overlapping timeframes were two before and two after the central frame as this allowed visualization of stable vortices that span over more than one time frame, differentiating that from noise and short-term fluid rotations. 3 areas were examined in the RV: 1- the main cavity of RV excluding the right ventricular outflow tract (RVOT), 2- the tricuspid inflow ring and 3- the RVOT. The spherical ROI was placed flush to the opening of the corresponding valve. The area of interest measurements was standardized and calculated in accordance with a normalization factor with the BSA to take into consideration the different sizes of the hearts between groups. Timing in the cardiac cycle was also recorded and standardized between cases so that the timeframes were matched while measuring the vorticity in all cases (Supplementary Video 1).

### Cardiac Modeling and RToF Atlas Building

Patient-specific biventricular geometries were generated using the Cardiac Image Modeler (CIM) (v8.3.0, University of Auckland, New Zealand) (20). Briefly, CIM is a semi-automatic segmentation software package developed to facilitate segmentation of both RV and LV endocardial and epicardial surfaces from all the short and long axis images. To generate patient-specific geometry, the user first identifies anatomical landmarks (RV insertion points, valve points and LV apex). These landmarks are then automatically tracked throughout the cardiac cycle using non-rigid registration and a 3D biventricular template is then fitted. Guide-points are then placed by the user to deform the model to those points in real-time and end diastolic volume (EDV), end systolic volume (ESV), stoke volume (SV) and ventricular mass are calculated by numerical integration. To identify abnormal patterns and study relationships between shape and clinical parameters, the rToF shape models were projected onto a biventricular atlas generated from 95 rTOF participants without history of valve replacement. The demographics of the atlas cases are shown in Table 1. The atlas was constructed using methods described previously (9, 10). Briefly, contours were drawn manually on both long axis and short axis slices by expert analysts using Segment (21). Interobserver errors are reported in (10). The biventricular shape model was then automatically customized to each patient using an iterative registration algorithm (9). Valve locations were customized to the manual landmarks by using landmark registration, and surfaces were customized by using diffeomorphic non-rigid registration to the manual contours. All the end diastolic (ED) mesh points were first aligned to the mean mesh surface points using Procrustes analysis to remove any pose variation (translation and rotation). This transformation was then applied to the end-systolic (ES) mesh. The ED and ES surface points were then concatenated to form a single combined shape and principal component analysis was then applied. The normalized scores of each mode reflected how much each individual differs from the population mean. Using a concatenation of ED and ES phases captured the variation in function as well as shape in the principal components.

**Table 1:** Demographics and cardiac parameters (mean ± s.d).

**Demographics**	**Volunteers** **(*n* = 10)**	**rToF** **(*n* = 12)**	**rToF Atlas** **(*n* = 95)**
Age (y)	32.4 ± 11.2	32.3 ± 11.2	19.5 ± 12.7^†^
Sex Male: Female	5:5	5:7	57:38
Height (cm)	173 ± 8.7	168.6 ± 8.6	154.7 ± 19.7^†^
Weight (kg)	78 ± 20.6	71 ± 12.2	58 ± 26.0^†^
BSA (m^2^)	1.9 ± 0.27	1.8 ± 0.15	1.5^†^
PRV (ml/cycle)	1.5 ± 1.70	17.1 ± 21.07^*^	20.8 ± 11.8
TRV (ml/cycle)	0.11 ± 0.34	1.06 ± 1.36^*^	NA
LV EDVi (ml/m^2^)	85.5 ± 17.2	90.9 ± 23.2	78 ± 14
LV SVi (ml/m^2^)	45.3 ± 8.15	44.2 ± 10.3	37.1 ± 8.0
LV mass index (g/m^2^)	60.1 ± 8.0	57.9 ± 5.1	76 ± 14^†^
RV EDVi (ml/m^2^)	92.8 ± 13.3	112.6 ± 20.4^*^	147 ± 14^†^
RV SVi (ml/m^2^)	41.3 ± 8.6	45.7 ± 14.8	52.9 ± 14.8^†^
RV mass index (g/m^2^)	30.5 ± 4.7	36.2 ± 12.7	42 ± 11
RV ESVi (ml/m2)	51.4 ± 7.3	66.8 ± 11.7^**^	90 ± 27^†^

* *p < 0.05*,

** *p < 0.01 rToF vs. volunteers*,

† *p < 0.05 rToF vs. rToF Atlas; BSA, body surface area; EDVi, indexed end diastolic volume; ESVi, indexed end systolic volume; LV, Left ventricle; PRV, pulmonary regurgitation volume; SD, standard deviation; SVi, indexed stroke volume; TR, tricuspid regurgitation; NA, not available*.

**Figure 1:**
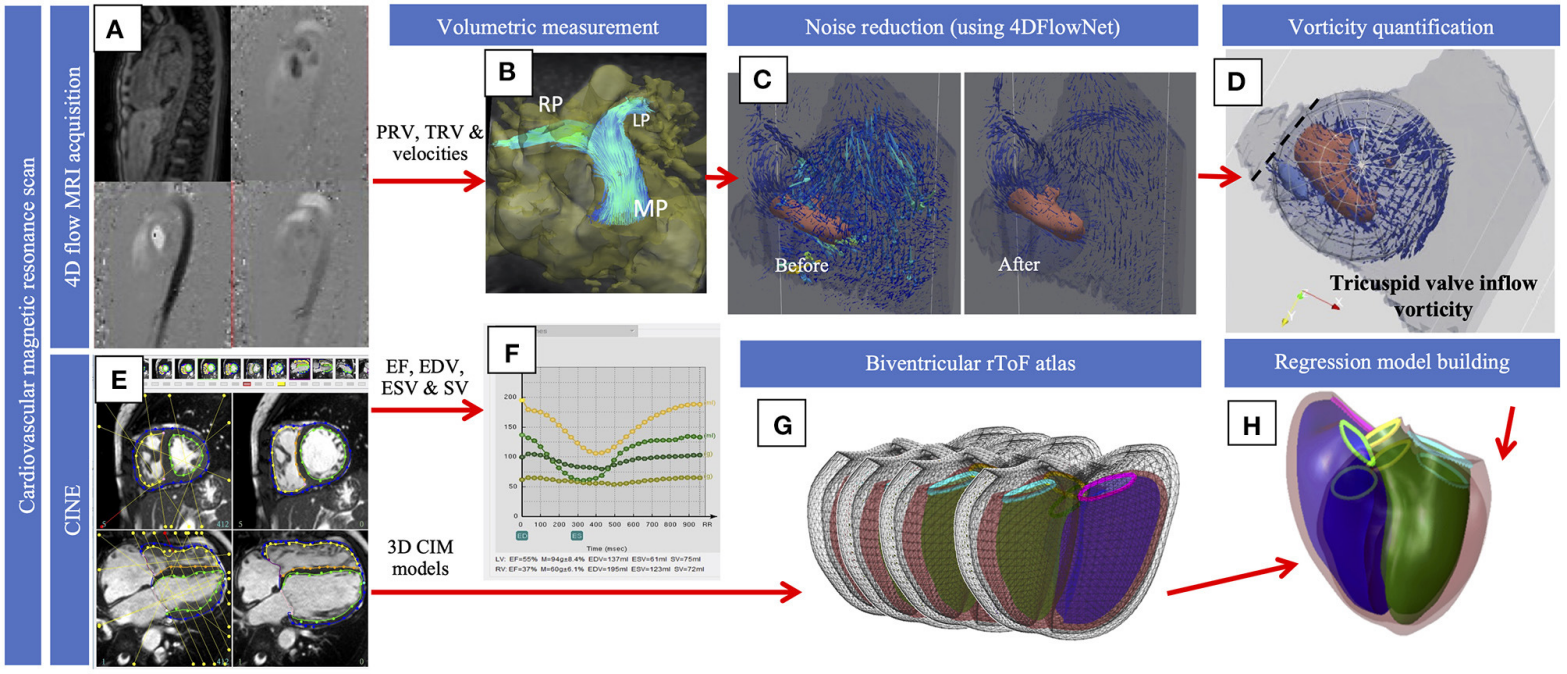
Diagrammatic representation of the methodology. 4D flow **(A)** was used for volumetric quantification of flow in the pulmonary and across the tricuspid valve **(B)** and velocity data was processed with the 4D flow neural network **(C)**. Vorticity **(D)** was quantified and visualized with the vortex core and streamlines that demonstrate velocity within the spherical ROI was shown, and the dotted line represents the opening of the tricuspid valve. Cines **(E)** were used to extract EDV, ESV and SV **(F)**. Cines were also used to build the models for the current population that were projected onto the atlas **(G)**. Regression models **(H)** were used to compute shape variations associated with vorticity. The figure shows an overlapping of the diagrams of a normal biventricular model and the model generated to show the effect of vorticity.

Patient-specific biventricular models were projected onto the first 24 rToF atlas shape modes which captured 90% of the total shape variation. Univariate regression was used to correlate principal component scores with cardiac indices and vorticity to identify shape associations. The main features of the modes with significant correlations were described in a similar manner to previous studies (9, 10). Multiple regression models were used to determine the association between biventricular shape and vorticity, controlling for effects of covariates sex, height, weight, and age. For the multiple regression models, the principal component scores were used as dependent variables and average vorticity and covariates (sex, height, weight, and age) were included as independent variables. A morphometric shape mode was then generated using the regression coefficient associated with vorticity, which quantifies the independent effect of vorticity on biventricular shape as described previously (9, 10). Diagrammatic demonstration of the methodology is shown in Figure 1.

### Statistics

Statistical analysis was performed using R (22) and SPSS software v. 25 (IBM SPSS, Chicago, IL). All continuous values are reported as mean ± standard deviation for continuous variables and as frequency for categorical variables. Statistical differences were presented by *p*-values using one-way ANOVA or Kruskal-Wallis test depending on the distribution. Differences were considered significant for *P* < 0.05. Bonferroni correction was used for multiple tests. All variables were standardized before regression.

## Results

### Demographics

12 rToF Patients and 10 Volunteers Were Included in the Study. Volunteers Were Used in Demographic and Vorticity Comparisons Only. Demographics and Cardiac Parameters Are Shown in Table 1 for Volunteers, Study Group, and Atlas Group. None of the rToF Patients Had Evidence of Pulmonary Stenosis.

### Quantitative Vortex Analysis

**Figure 2:**
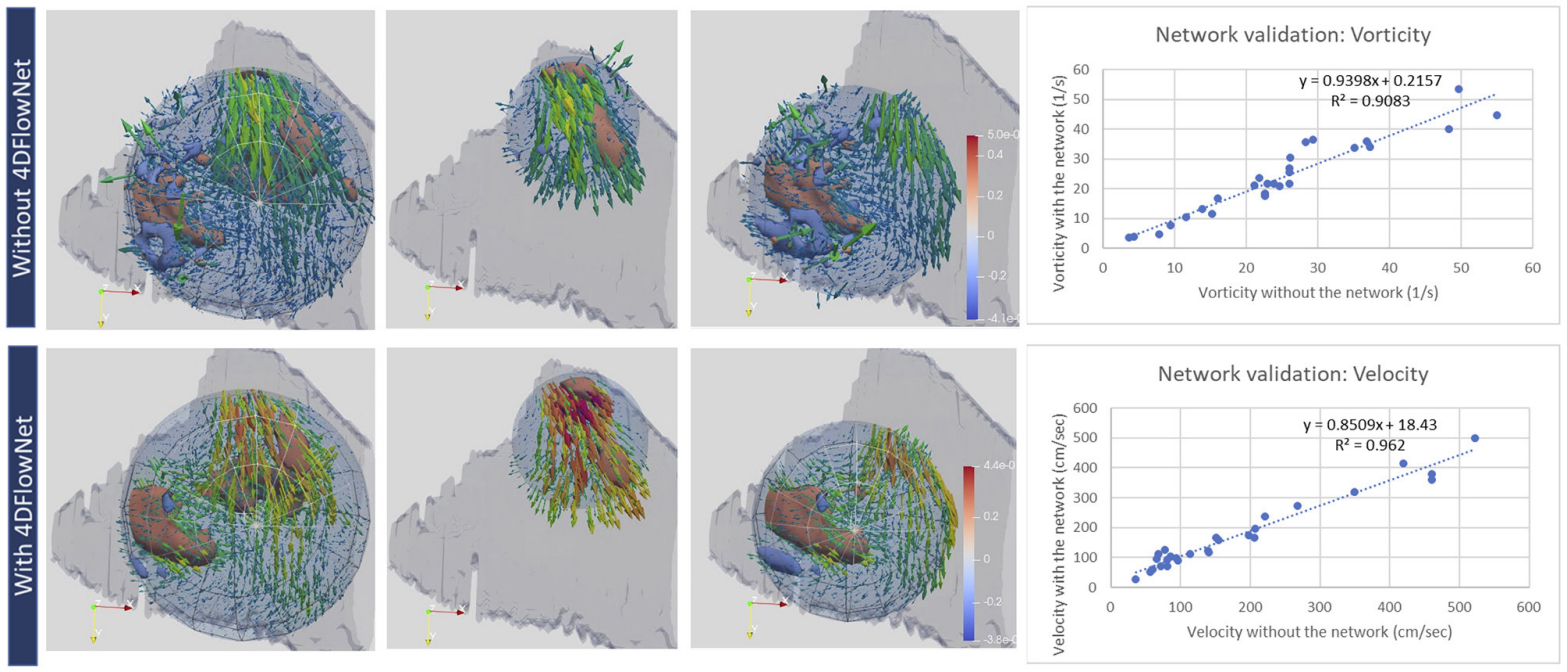
Qualitative (left) and quantitative (right) evaluation of velocity and vorticity with and without the 4DFlownet. The panels show the 3 regions of interest where vorticity was captured (left: whole RV; middle: RVOT; right: tricuspid inflow in diastole. The network reduced noise making the vorticity structure more readily visualized. Right plots show mean vorticity calculated in the region of interest and mean velocity calculated at planes positioned in the RVOT, showing high correlation before and after 4DFlowNet.

Isolation of the RV was done with a spherical region of interest considering all the main flow areas. However, some flow outside the ventricle was inevitably included. The amount of error accounted for an average of 1% of the vorticity that was measured, which was considered negligible. This was validated through placement of regions inside the ventricles with exact boundary adjustment, then extension of the boundaries beyond these boundaries to include areas that did not show flow and the difference was calculated. The effect of 4D Flow network in reducing noise is shown in Figure 2. Vortex structure was more readily visualized after enhancement, while average vorticity values were in high agreement pre and post enhancement. Table 2 shows average vorticity in our two groups at different areas of interest.

**Table 2:** Vorticity in the volunteer and study groups.

	**Volunteer**	**rToF**	*T*-test^*^
RV vorticity^**^	23.5 ± 6.5	25.5 ± 5.9	0.59
Tricuspid vorticity	23.5 ± 6.5	20.6 ± 5.4	0.35
RVOT vorticity	5.12 ± 1.9	10.4 ± 5.5	0.006

* *t-test is significant at P < 0.05*,

** *vorticity is measured in (1/s)/100. Vorticity was measured across multiple timeframes and averaged over the diastolic phase*.

### RV Shape Scores

The absolute point-to-point error between the shapes generated by the 24 shape mode scores and the original shapes was 3.4 ± 0.6 mm at ED and 3.6 ± 0.5 mm at ES, showing that the atlas shape scores formed a good approximation to the study group geometry. The differences in volume were: LVEDV: 4.3 ± 6.1 mL; RVEDV: 2.4 ± 6.2 mL; LVESV:−0.7 ± 4.2 mL; RVESV:−2.2 ± 4.2 mL and the mass differences were: LVM:−3.5 ± 4.9 g; RVM: 0.7 ± 3.5 g (original minus atlas, mean ± s.d.).

### RV Shape and PRVi

**Figure 3:**
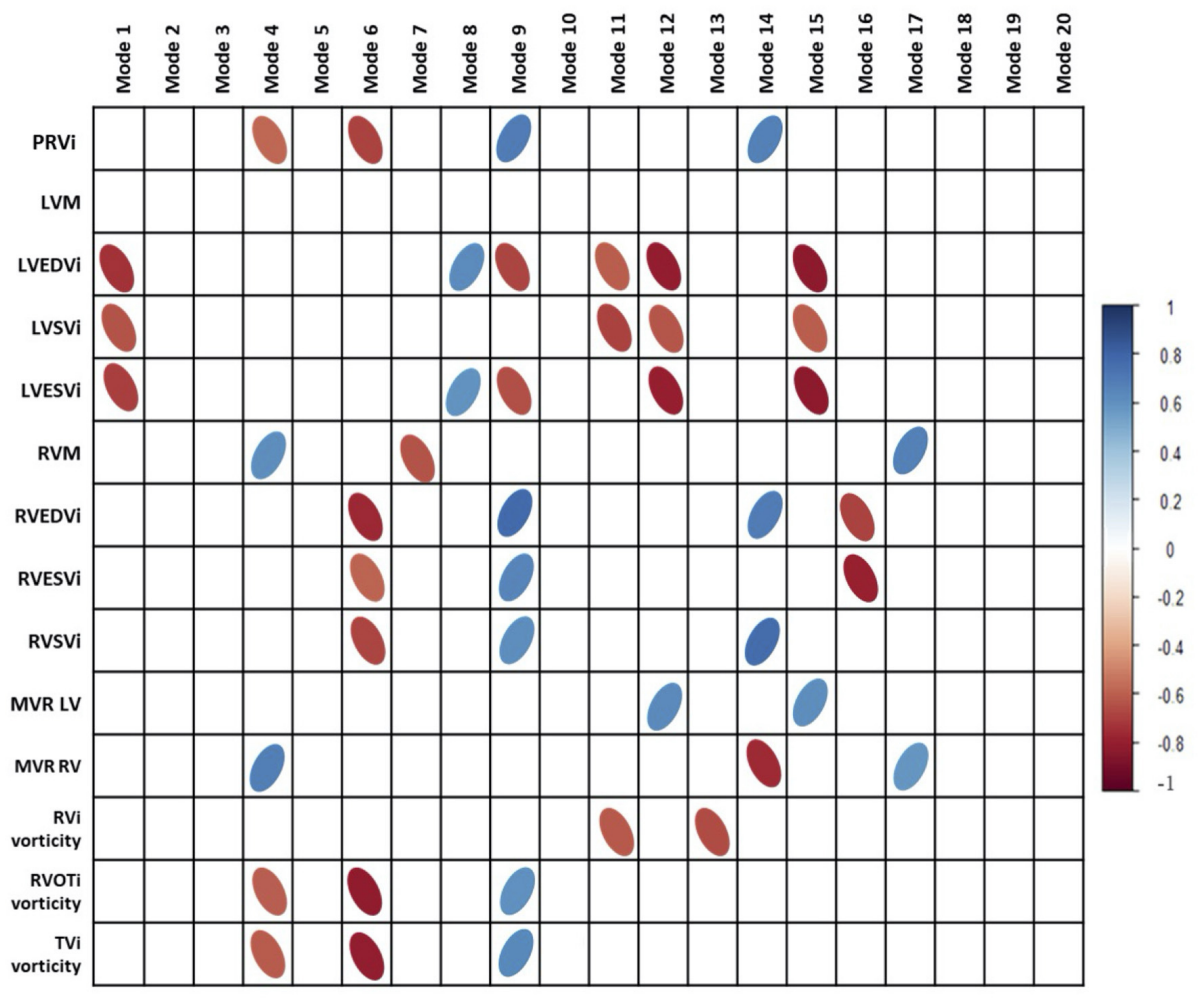
Correlations of cardiac indices and vorticity with principal component analysis modes. PRVi, indexed pulmonary regurgitant volume; LVM, left ventricular mass; LVEDVi, indexed left ventricular end diastolic volume; LVSVi, indexed left ventricular stroke volume; LVESVi, indexed left ventricular end systolic volume; MVR, mass to volume ratio. Modes 9 and 6 Are the most correlated modes with aspects of cardiac metrics and vorticity. Bonferroni correction was done with all correlations.

**Figure 4:**
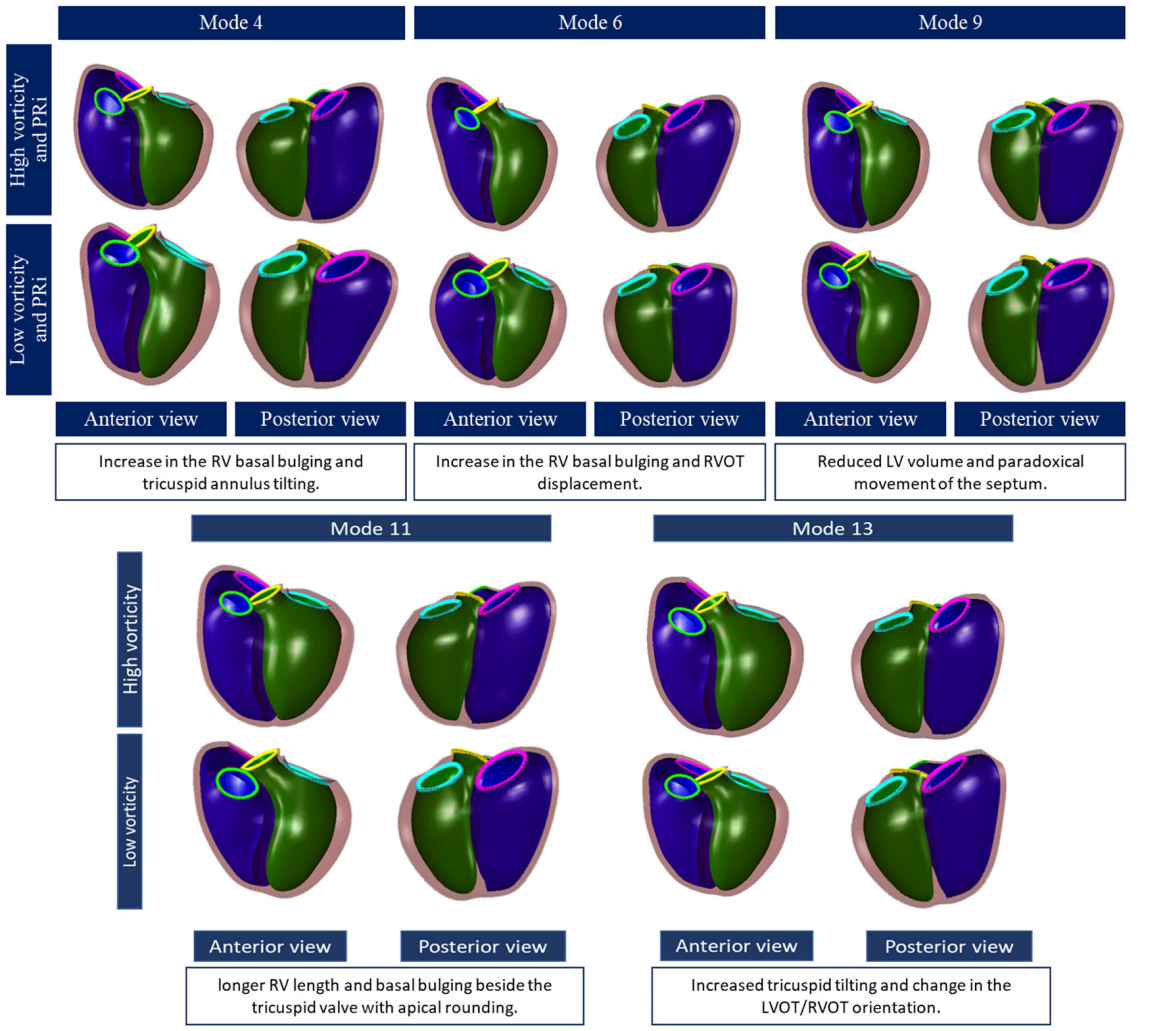
Principal component shape variations associated with pulmonary regurgitant volume (top) and vorticity (bottom). The RV is shown in purple, left ventricle: green, the tricuspid valve: pink, the pulmonary valve: neon green, the aortic valve: yellow and mitral valve: blue. Each mode is shown in an anterior and posterior view with description below.

PRVi showed significant correlation with mode 4, mode 6 and mode 9 (*r* = −0.55, *r* = −0.50 and *r* = 0.6 respectively, *p* < 0.05). Correlations between shape scores and more cardiac metrics are shown in Figure 3. Figure 4 shows the visualization and description of modes associated with PRVi. Greater PRVi was associated with RV dilatation, with an increase in the basal bulging, apical bulging, and an increase in tricuspid annulus tilting. The LV size was reduced with paradoxical movement of the septum toward the RV during systole. RV EDVi, ESVi and SVi correlated with modes 6 and 9, whereas the association with LV size was reversed with respect to mode 9, consistent with reduced LV volume with increased PRVi.

### RV Shape and Vorticity

Indexed RV outflow and indexed tricuspid inflow vorticity were both correlated with the same three modes associated with PRVi: mode 4 (*r* = −0.63 and *r* = −0.6 respectively), mode 6 (*r* = −0.82 and *r* = −0.78 respectively) and mode 9 (*r* = 0.6 and *r* = 0.62 respectively). Inflow and outflow vorticity were therefore also associated with an increase in the RV basal bulging, RV apical bulging and tricuspid annulus tilting. Correlations of vorticity and shape scores are shown in Figure 3.

In contrast, vorticity over the whole RV was correlated with modes which were not correlated with PRVi. Maximum and average RV vorticity correlated with mode 11 (*r* = −0.62 and *r* = −0.63 respectively, *p* < 0.05, Figure 4) indicating that higher vorticity was associated with longer ventricular length, bulging beside the tricuspid valve with some abnormal tricuspid tilting. Average RV vorticity was correlated with mode 13 (*r* = −0.69, *p* < 0.05), associated with an increase in tricuspid tilt (Figure 4). Through these modes, increased vorticity was associated with increased LV size, rather than the reduction in LV size seen in the modes associated with PRVi.

### Multivariate Analysis

**Figure 5:**
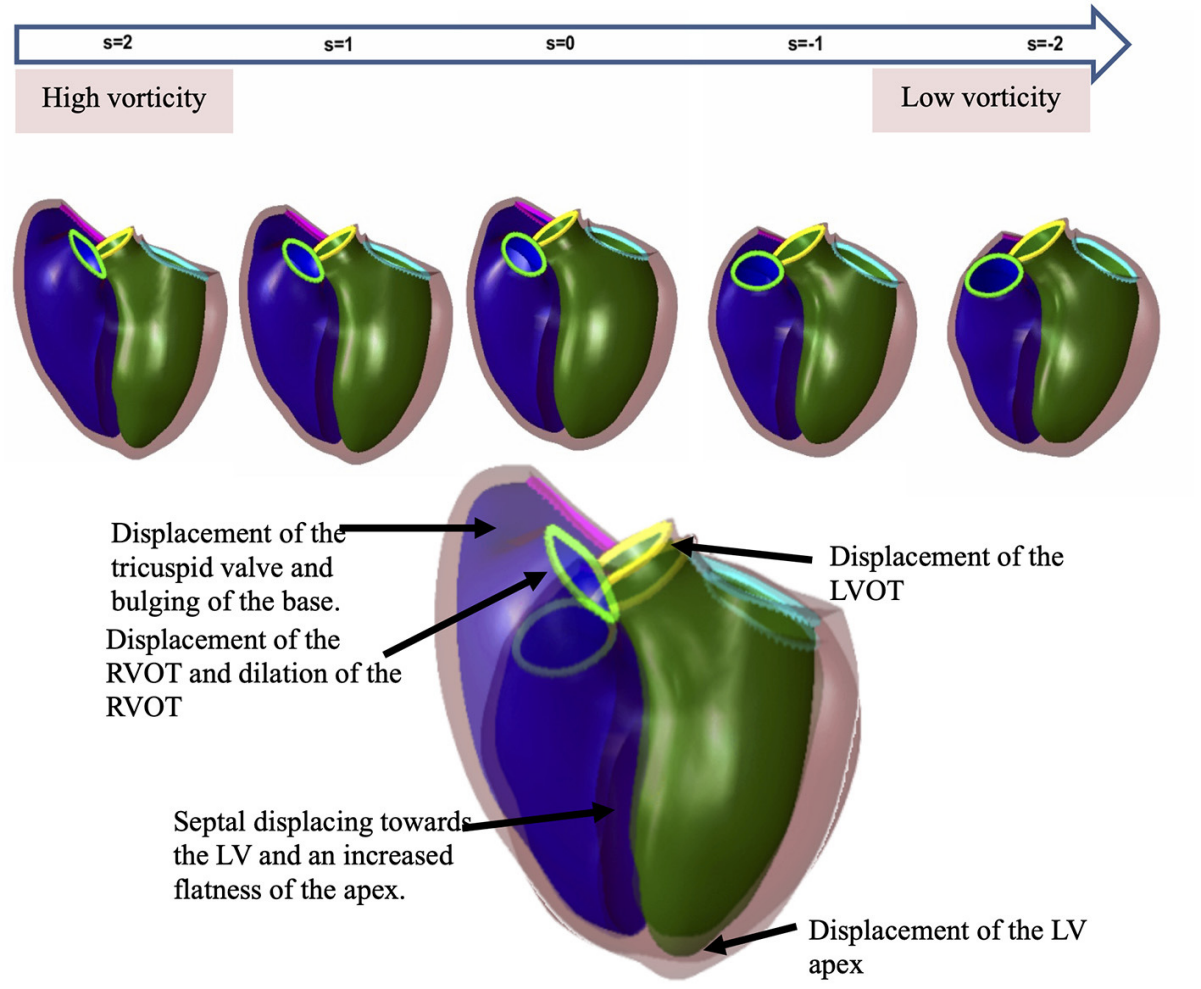
The effect of vorticity on the shape of the RV by a regression model. The spectrum of shapes is demonstrated in the line of shapes above to show the difference between a normal biventricular model (far right) and a model affected by high vorticity (far left). The larger overlapping shapes emphasize the differences. The RV enlargement affects the orientation of both the pulmonary and tricuspid valves. There is an architectural shift from the normal configuration to an enlarged bulging base. There is an evident effect on the LV as well showing the change in wall shape and position of the aortic and mitral valves due to the shift in the RV outflow tract configuration.

Figure 5 shows the results of the multivariate analysis. The model demonstrated the relationship between average vorticity and biventricular shape while controlling for the effects of sex, height, weight and age. The RV enlargement affected the orientation of both the pulmonary outflow tract and tricuspid valves. There was an architectural shift from the normal configuration to an enlarged bulging base. There was an evident effect on the LV as well, showing lengthening of the LV, and change of position of the aortic and mitral valves due to the shift in the RVOT configuration. Increased RV vorticity was associated with an increase in LV size (Supplementary Video 2).

## Discussion

This study, to the best of our knowledge, is the first to examine RV shape associations with flow vorticity in patients with rToF using statistical shape analysis. A statistical atlas of rToF shape variation was used as a reference to describe shape with relatively few parameters (9, 23, 24). These population-based atlases enable characterization of ventricular shape in a targeted patient or group of related patients. This method enabled evaluation of the change in the regional ventricular anatomy rather than the volume as a whole (5). Individual patient status was quantified using mode scores which showed good agreement with the actual geometry. We found that inflow and outflow RV vorticity was significantly correlated with shape modes which were also significantly correlated with PRVi. However, total RV vorticity was significantly correlated with shape modes which were independent of PRV. These preliminary results suggest that mechanisms of ventricular remodeling may include intra-ventricular flow vorticity.

The application of deep neural network image enhancement to intraventricular vorticity estimation is promising. We found that the network, although trained on computational fluid dynamics simulations in patient specific aortic flow regimes, was able to denoise intraventricular 4D flow data due to its ability to recognize coherent flow patterns. The visualization of the vortex structure was considerably improved, while the average vorticity estimate was not affected (Figure 2).

The RV dilation pattern associated with PRVi was observed as an apical and basal bulging. The outlet also dilated with apical deformation as regurgitation became worse. In addition to RV changes, there was septal paradoxical movement toward the RV and a change in the left ventricular architecture as well. These results agree with previous atlas-based analyses (3, 6) which used 2D flow MRI and color doppler measurement to quantify PVR. 4D flow MRI has been shown to be more accurate for this task (25, 26) leading to higher correlation coefficients in our study compared with (3).

Correlation of vorticity with deformation modes were significant with the same modes that correlated with PRVi, with similar or higher correlation, confirming the interaction between vorticity, PRVi and shape. However, total RV vorticity correlated with 2 modes that did not correlate with PRVi (modes 11 and 13). This suggest that higher total vorticity is also associated with longer ventricular length. Also an increase bulging beside the tricuspid valve was observed with some abnormal tricuspid tilting. This is an interesting finding given that our study group had low to mild tricuspid regurgitation. Both these modes did not correlate with PRVi which indicated that these changes are independent of the amount of PRVi suggesting that the adverse vorticity in the whole ventricle was the influencer of change with these 2 modes. A study on the pulmonary artery (19) suggested that with the dilation of the vessel and a compromised RV function, blood creates shear layers, with differences in the velocity of each layer resulting in the higher vorticity formation. If this is applied to the dilated ventricle, a chaotic flow caused by pulmonary regurgitation would result in an abnormal vorticity affecting the architecture. This would suggest that the consequence of flow variation can have separate effects to the degree of regurgitation. In a study on vorticity in the RV (18) it was suggested that altered vorticity may be a cause for ventricular energy loss and RV dilatation. This was supported by another study (27) on ventricular kinetic energy which indicated a positive correlation between EDV and turbulent kinetic energy. The same study observed the change in kinetic energy to extend toward the apex, not only in the RV outflow tract, which is in line with our findings on vorticity that differs in behavior between the whole RV compared with inflow and outflow regions. This in turn could explain that abnormal fibrosis induced by changes in flow could affect places that are independent of the degree of pulmonary regurgitation.

There was a correlation between vorticity and modes that described a rounder apex. This could be explained by minimal apical flow observed in volunteers (18) while in patients, marginal flow was a finding and heterogeneous vortex formations were observed. Our study confirms this visual finding by the high correlation with modes that indicated a wider apex. The apical changes had been previously observed by another study (6) that suggested that the apical trabeculations contribute to how the RV adapts to volume overload in rToF. This is also in agreement with previous studies that observed a flatter apex (7, 28).

Our study also shed light on associations between vorticity and the architecture of the RV outflow tract. The pulmonary annulus was displaced anteriorly, and this decreased the concavity that is between the tricuspid valve and the pulmonary valve in mode 6, which was correlated with PRVi and vorticity in the outflow tract. This is in agreement with previous studies that observed a significantly more convex RV outflow tract in rToF (5, 6). The correlation of mode 6 with vorticity and PRVi suggest different remodeling mechanisms than those relating curvature change to postsurgical scarring (29). Furthermore, RV EDVi correlated with mode 6 as well, confirming the relation between volume overload and the outflow tract shape change.

The normal septum is convex toward the right ventricle with the left being a thicker rounder ventricle (30). The systolic motion of the septum to the left happens while the normal configuration is maintained. However, it has been observed in previous studies in patients with right ventricular volume overload that the septum flattens and the convexity may be reversed to be toward the left (30, 31). Vorticity was correlated with mode 4 which expressed the septal paradoxical motion. This is in agreement with previous studies that described the septal curvature (32).

### Limitations

In this pilot study, our results are limited by a small sample size of patients examined with 4D Flow. RV shape analysis and vorticity extraction relied on segmentation and manually placed areas of interest, which may be automated in the future using machine learning methods. The patients in the atlas were younger than those in the study group, which may lead to differences in shape. However, the shape modes were able to describe the patient-specific geometry with good accuracy, since the atlas covers a wide range of developing disease states. Since we customized the atlas scores to each patient, the mismatch between atlas and 4D flow patients is not critical. Information on type of repair, residual RV obstruction and patient history were not available. More prospective, larger scale, multicenter studies are required to assess the validity and effectiveness of this methodology.

## Conclusions

RV and LV shape features were significantly related to vorticity, and some of these relationships were not explainable by pulmonary regurgitant volumes or volumetric variations alone. Flow vorticity therefore may provide additional mechanistic information about remodeling and developing disease in rToF patients. Both LV and RV shape are important in understanding rToF RV flow vorticity patterns.

## Data Availability Statement

The raw data supporting the conclusions of this article will be made available by the authors, without undue reservation.

## Ethics Statement

The studies involving human participants were reviewed and approved by Health and Disability Ethics Committee New Zealand (17/CEN/226). The patients/participants provided their written informed consent to participate in this study.

## Author Contributions

AE collated the data and performed the analyses. All authors participated in concept and design, revision, and final approval of the submitted manuscript.

## Funding

This work was supported by the New Zealand Heart Foundation, NIH R01HL121754 from the National Heart, Lung, and Blood Institute USA, the Health Research Council of New Zealand grants 17/234 and 17/608, core funding from the Wellcome/EPSRC Centre for Medical Engineering [WT203148/Z/16/Z], and a grant from Siemens Healthineers, Erlangen, Germany.

## Conflict of Interest

This study received working expenses and in-kind support from Siemens Healthineers, Erlangen, Germany. The funder was not involved in the study design, collection, analysis, interpretation of data, the writing of this article or the decision to submit it for publication. AM and JHO are co-founders of and have an equity interest in Insilicomed, Inc., and serves on the scientific advisory board. Some of their research grants, including those acknowledged here, have been identified for conflict of interest management based on the overall scope of the project and its potential benefit to Insilicomed, Inc. The authors are required to disclose this relationship in publications acknowledging the grant support, however the research subject and findings reported here did not involve the company in any way and have no relationship whatsoever to the business activities or scientific interests of the company. The terms of this arrangement have been reviewed and approved by the University of California San Diego in accordance with its conflict of interest policies. The remaining authors declare that the research was conducted in the absence of any commercial or financial relationships that could be construed as a potential conflict of interest.

## Publisher's Note

All claims expressed in this article are solely those of the authors and do not necessarily represent those of their affiliated organizations, or those of the publisher, the editors and the reviewers. Any product that may be evaluated in this article, or claim that may be made by its manufacturer, is not guaranteed or endorsed by the publisher.

## Supplementary Material

Supplementary Video 1 Video demonstrating the workflow of vorticity visualization and quantification. 4d Flow velocity data is quantified with Siemens 4D flow software then denoised with 4DFlowNet then visualized on ParaView. CIM is used to create a mask that is superimposed on the velocity data. The final visualization (far right) shows the final result which is visualized with the velocity vector fields, and vorticity cores.

Supplementary Video 2 The regression model demonstrates each stage of shape evolution during ED and ES with the increase of vorticity and how much it changes the biventricular shape. The shape evolves from normal to what we observe with higher vorticity.
